# Neurological comorbidity and severity of COVID-19

**DOI:** 10.1007/s00415-020-10123-y

**Published:** 2020-08-04

**Authors:** Alberto Romagnolo, Roberta Balestrino, Gabriele Imbalzano, Giovannino Ciccone, Franco Riccardini, Carlo Alberto Artusi, Marco Bozzali, Bruno Ferrero, Elisa Montalenti, Elisa Montanaro, Mario Giorgio Rizzone, Giovanna Vaula, Maurizio Zibetti, Leonardo Lopiano

**Affiliations:** 1 Department of Neuroscience “Rita Levi Montalcini”, University of Turin, Via Cherasco 15, 10126 Turin, Italy; 2 Section of Clinical Epidemiology, Center for Epidemiology and Oncologic Prevention (CPO Piemonte), Città della Salute e della Scienza, Turin, Italy; 3 Department of Medical Sciences, Front Line P.S. P.O. Molinette, Città Della Salute e Della Scienza, Turin, Italy; 4 Department of Neuroscience, Brighton& Sussex Medical School, University of Sussex, Brighton, East Sussex UK

**Keywords:** COVID-19, Neurology, Stroke, Cerebrovascular disease, Dementia

## Abstract

**Objective:**

Neurological symptoms of COVID-19 patients have been recently described. However, no comprehensive data have been reported on pre-existing neurological comorbidities and COVID-19. This study aims at evaluating the prevalence of neurological comorbidities, and their association with COVID-19 severity.

**Methods:**

We evaluated all consecutive patients admitted to the Emergency Room (ER) of our hospital between the 3rd March and the 14th April 2020, and diagnosed with COVID-19. Data on neurological and non-neurological diseases were extracted, as well as data on demographic characteristics and on severity degree of COVID-19. The prevalence of neurological comorbidities was calculated, and multivariate binary logistic regression analyses were used to estimate the association between neurological diseases and COVID-19 severity.

**Results:**

We included 344 patients. Neurological comorbidities accounted for 22.4% of cases, with cerebrovascular diseases and cognitive impairment being the most frequent. Neurological comorbidity resulted independently associated with severe COVID-19 (OR 2.305; *p *= 0.012), as well as male gender (*p *= 0.001), older age (*p *= 0.001), neoplastic diseases (*p *= 0.039), and arterial hypertension (*p *= 0.045). When neurological comorbidity was associated with non-neurological comorbidities, the OR for severe COVID-19 rose to 7.394 (*p *= 0.005). Neurological patients, in particular cerebrovascular and cognitively impaired ones, received more respiratory support indication.

**Conclusion:**

Neurological comorbidities represent a significant determinant of COVID-19 severity, deserving a thorough evaluation since the earliest phases of infection. The vulnerability of patients affected by neurological diseases should suggest a greater attention in targeting this population for proactive viral screening.

## Introduction

Coronavirus disease 2019 (COVID-19) is an infectious disease caused by severe acute respiratory syndrome coronavirus-2 (SARS-CoV-2), declared a pandemic on 11 March 2020 [1]. Clinical features range from the absence of symptoms to severe respiratory failure [2]. A rapidly increasing number of articles have been published on COVID-19, including numerous reports and studies on associated neurological symptoms and complications [3], such as acute stroke [4], hyposmia [5], Guillain–Barrè syndrome [6], encephalitis [7]. It has been shown that about one-third of COVID-19 patients develop neurological symptoms [3, 8], in most cases associated with a more severe infection [3], indicating a potential neurotropism of SARS-CoV-2 as one of the possible mechanisms of neurological damage [9, 10]. A recent retrospective study reported that inpatients from a neurological ward affected by COVID-19 showed a worse outcome compared to those infection-free [11]. However, no data have been reported yet on the prevalence and the association with infection severity of pre-existing neurological comorbidities in COVID-19 patients. A scoping review on the occurrence of neurological diseases in COVID-19 patients reported a prevalence of about 8% [12]. Nevertheless, this review highlighted the methodological heterogeneity in the reviewed papers, which limit a reliable estimation of prevalence of neurological diseases in COVID-19 patients, as well as their association with the infection severity.

The aim of our study was to evaluate, on a large cohort of consecutive patients admitted to the Emergency Room (ER) and diagnosed with COVID-19, the prevalence of neurological comorbidities and their possible association with a more severe form of COVID-19 onset.

## Methods

We evaluated all consecutive patients admitted to the ER of the “Città della Salute e della Scienza di Torino Hospital” between 3 March 2020 and 14 April 2020, and diagnosed with COVID-19 by means of a positive Reverse Transcription Polymerase Chain Reaction nasopharyngeal swab.

### Comorbidities definition

In all patients, we assessed both neurological and non-neurological comorbidities. A medical condition was attributed to the patients when: (a) defined diagnosis, and/or (b) unequivocal diagnostic test results, and/or (c) specific medical/surgical treatment, and/or (d) specific follow-up were reported. Moreover, previous clinical notes available in our hospital electronic archives were reviewed.

The Charlson Comorbidity Index (CCI) [13], defined as the sum of the weighted scores of various comorbid conditions (Table 1), was calculated for each patient to grade their comorbid conditions.

**Table 1:** Components and relative weights of the Charlson Comorbidity Index

Weight	Comorbidities
1 ×	Myocardial infarction
	Congestive heart failure
	Peripheral vascular disease
	Cerebrovascular disease
	Dementia
	Chronic obstructive pulmonary disease
	Connective tissue disease
	Ulcer disease
	Mild liver disease
	Diabetes mellitus
2 ×	Hemiplegia
	Moderate/severe renal disease
	Diabetes with end-stage organ damage
	Any tumor
	Leukemia
	Lymphoma
3 ×	Moderate/severe liver disease
6 ×	Metastatic solid tumor
	AIDS

The Charlson Comorbidity Index results by the sum of the various comorbidities multiplied by their weight score

### Infection severity definition

The severity of COVID-19 at the time of admission to ER was evaluated by means of the 2007 Infectious Diseases Society of America/American Thoracic Society Criteria for Defining Severe Community-acquired Pneumonia [14]. Severe disease was identified in patients presenting with one major criterion or three or more minor criteria (Table 2).

**Table 2:** 2007 Infectious Diseases Society of America/American Thoracic Society Criteria for Defining Severe Community-acquired Pneumonia

Major criteria
Septic shock with need for vasopressors
Invasive mechanical ventilation

Minor criteria
Respiratory rate ≥ 30 breaths/min
PaO2/FIO2 ratio ≤ 250
Multilobar infiltrates
Confusion/disorientation
Uremia (BUN level ≥ 20 mg/dl)
Leukopenia as a result to infection alone (WBC count < 4,000 cells/ml)
Thrombocytopenia (platelet count < 100,000/ml)
Hypothermia (core temperature < 36° C)
Hypotension requiring aggressive fluid resuscitation

A severe form of infection was defined by the presence of either one major criterion or three or more minor criteria

*BUN* blood urea nitrogen; *PaO2/FiO2* arterial oxygen pressure/fraction of inspired oxygen, *WBC* white blood cell

### Discharge from ER definition

Patients’ discharge from the ER was classified as follows

- Home: discharge at home with indication to fiduciary isolation and health surveillance by the general practitioner;
- Internal Medicine Unit without mechanical respiratory support: admission to a ward equipped for best medical treatment and respiratory support with oxygen mask;
- Internal Medicine Unit with non-invasive mechanical respiratory support: admission to a ward equipped with non-invasive mechanical ventilation for respiratory support;
- Intensive Care Unit (ICU): admission to a ward equipped with invasive mechanical ventilation for respiratory support.

### Statistical analysis

Descriptive statistics (mean, standard deviation, range) were used for continuous variables and frequency for categorical data. The Mann–Whitney test and Fisher’s exact test were used for comparisons between groups, as appropriate. A first univariate binary logistic regression was used to estimate the odds ratio (OR) of presenting severe infection (dependent variable), considering as independent variables various demographic and clinical features, including neurological comorbidity. Then, we performed a second, multivariate, binary logistic regression analysis considering as independent variables every feature resulting significantly associated with severe infection in the previous univariate analysis. Finally, a third binary logistic regression analysis was performed, adjusting for age and CCI, considering four categories of patients as independent variable: (a) absence of any neurological or non-neurological disease (used as control group) (b) presence of neurological disease without other comorbidities, (c) presence of neurological disease plus other comorbidities, and (d) presence of comorbidities without neurological diseases. The Hosmer and Lemeshow’s goodness-of-fit test was applied. In case of multiple comparisons, Bonferroni’s correction was used. All *p*-values reported were two-tailed, and a *p *< 0.05 was considered statistically significant. Data were analyzed using the Statistical Package for the Social Sciences (SPSS 22 for Mac, Chicago, IL).

### Ethics

This study received approval from the ethical standards committee on human experimentation (*Comitato Etico Interaziendale AOU Città della Salute e della Scienza di Torino, AO Ordine Mauriziano di Torino, ASL Città di Torino*; Protocol number 00172/2020, approved May 5th, 2020), and patients gave their written informed consent.

## Results

The main demographic and clinical features of 344 consecutive patients evaluated are summarized in Table 3.

**Table 3:** Demographic and Clinical features

Gender (males/females)	204/140 (59.3%/40.7%)
Age (years)	61.5 ± 17.8 (15-98)
Time from symptoms onset (days)	6.1 ± 4.4 (0-30)
Symptoms at onset (%)	
Fever	100 (29.1%)
Cough	32 (9.3%)
Dyspnea	17 (4.9%)
Anosmia	7 (2.0%)
Myalgia/Asthenia	6 (1.7%)
Sore throat	5 (1.5%)
Confusion	4 (1.2%)
Thoracic pain	3 (0.9%)
Diarrhea	2 (0.6%)
Conjunctivitis	1 (0.3%)
Jaundice	1 (0.3%)
Syncope	1 (0.3%)
Fever and cough	88 (25.6%)
Fever and dyspnea	13 (3.8%)
Fever and diarrhea	10 (2.9%)
Fever and anosmia	5 (1.5%)
Cough and dyspnea	5 (1.5%)
Fever and sore throat	4 (1.2%)
Three or more symptoms	40 (11.6%)
Neurological Diseases—*N* (%)	77 (22.4%)
Arterial Hypertension—*N* (%)	158 (45.9%)
Neoplastic Diseases—*N* (%)	49 (14.2%)
Diabetes—*N* (%)	42 (12.2%)
Chronic obstructive pulmonary disease—*N* (%)	41 (11.9%)
Renal failure—*N* (%)	18 (5.2%)
Charlson Comorbidity Index	2.0 ± 2.5 (0-16)
Institutionalized patients—*N* (%)	19 (5.5%)
Severe infection—*N* (%)	118 (34.3%)
Discharge from Emergency Room—*N* (%)	
Home	126 (36.6%)
Internal Medicine Unit without mechanical respiratory support	170 (49.4%)
Internal Medicine Unit with non-invasive mechanical respiratory support	25 (7.3%)
Intensive Care Unit	23 (6.7%)

Results are reported as average ± standard deviation (*range*) or absolute values (percentage), as appropriate

The mean age of the entire sample was 61.5 years: 4.7% of patients were ≤30-year-old, while 33.7% were ≥70-year-old. Male accounted for 59.3% of cases. The mean latency between the symptoms’ onset and the ER admittance was 6.1 days, ranging from 0 to 30 days; only two patients (0.6%) with mild symptoms (sore throat and sporadic cough) had a latency of 30 days, while 91.6% of patients were evaluated within 10 days from the onset, and 7.8% between 11 and 15 days. The most frequent symptom at COVID-19 onset was fever (74.1%; *n *= 255; isolated in 100 patients, associated with ≥1 other symptoms in 155 patients), followed by cough (44.8%; *n *= 154), dyspnea (13.7%; *n *= 47), and diarrhea (9.3%; *n *= 32). The majority of patients reported a monosymptomatic onset (52.0%; *n *= 179), while 36.3% (*n *= 125) presented with two symptoms, and 11.6% (*n *= 40) with three or more symptoms. Thirty-four percent of cases (*n *= 118) entered the ER with a severe form of infection.

Arterial hypertension was the most frequent comorbidity (45.9%), followed by neurological diseases (22.4%), neoplastic diseases (14.2%), diabetes (12.2%), chronic obstructive pulmonary disease (COPD) (11.9%), and renal failure (5.2%).

None of the patients were treated with specific antiviral drugs before admission; 9 of them (2.6%) were on hydroxychloroquine therapy (200 mg bid; therapy duration 5.2 ± 2.6 days, range 1–10).

### Prevalence and type of neurological diseases

A total of 22.4% of patients (*n *= 77) showed a neurological comorbidity. Compared to patients not affected by neurological diseases (Table 4), they were disproportionately overrepresented among the severe COVID-19 (*p *< 0.001). They were older (mean difference 16.2 years; p < 0.001), had a shorter interval between symptoms onset and ER admittance (mean difference 1.9 days; *p *= 0.001), and were more frequently affected by hypertension, renal failure, and neoplastic diseases (*p*≤0.001); their CCI was higher (*p *< 0.001), and they presented a higher prevalence of institutionalization (*p *< 0.001). Pre-existing cerebrovascular diseases were the most common comorbidity, affecting 39.0% of patients (*n *= 30/77, including 7 patients with hemorrhagic and 23 with ischemic stroke), followed by cognitive impairment (32.5%, including 4 patients with Mild Cognitive Impairment, 10 with Alzheimer’s Disease or Alzheimer’s Disease-like dementia, and 11 with vascular dementia), migraine or chronic tension-type headache or trigeminal neuralgia (14.3%; *n *= 11), epilepsy (6.5%; *n *= 5), peripheral neuropathy (5.2%; *n *= 4), Parkinson disease (1.3%; *n *= 1), and multiple sclerosis (1.3%; *n *= 1).

**Table 4:** Differences between patients with or without neurological disease

Demographic and Clinical features	Patients with neurological disease (*N *= 77)	Patients without neurological disease (*N *= 267)	P value
Severe infection – N (%)	51 (66.2%)	67 (25.1%)	< .001
Gender (males/females - %)	37/40 *(48.1%/51.9%)*	167/100 *(62.5%/37.5%)*	0.023
Age (years)	74.1 ± 15.9 *(30*-*97)*	57.9 ± 16.6 *(15*-*98)*	< .001
Time from symptoms onset (days)	4.6 ± 3.8 *(0*-*15)*	6.5 ± 4.5 *(0*-*30)*	0.001
Arterial Hypertension – N (%)	57 (74.0%)	101 (37.8%)	< .001
Neoplastic Diseases – N (%)	23 (29.9%)	26 (9.7%)	< .001
Diabetes – N (%)	12 (15.6%)	30 (11.2%)	0.305
Chronic Obstructive Pulmonary Disease—*N* (%)	13 (16.9%)	28 (10.5%)	0.127
Moderate-to-severe renal failure—*N* (%)	10 (13.0%)	8 (3.0%)	0.001
Charlson comorbidity index	4.3 ± 2.9 *(0*-*16)*	1.3 ± 1.8 *(0*-*11)*	< .001
Institutionalized patients—*N* (%)	15 (19.5%)	4 (1.5%)	< .001

Results are reported as average ± standard deviation (*range*) or absolute values (percentage), as appropriate. *p* value: statistical comparison between the two groups

A minority of patients (18.2%; *n *= 14) suffered only from neurological disease. The remaining 63 patients suffered also from one (32.5%; *n *= 25), two (37.7%; *n *= 29), or three or more (11.7%; *n *= 9) other comorbidities, with arterial hypertension representing the most frequent comorbidity (90.5% of cases), followed by neoplastic diseases (36.5% of cases).

### Neurological diseases and infection severity

The univariate binary logistic regression analysis (Table 5) revealed that a more severe form of infection was significantly associated with the presence of neurological disease (OR 5.855; 95% CI 3.387–10.122; *p *= 0.001), together with male gender, arterial hypertension, diabetes, renal failure, COPD, neoplastic disease, institutionalization, older age, and higher CCI.

**Table 5:** Univariate analysis of association between patients’ characteristics and severe form of infection

Demographic and clinical features	OR (95% CI)	P value
Male gender	1.942 (1.212–3.110)	0.006
Age	1.076 (1.057–1.095)	< .001
Time from symptoms onset	0.998 (0.949–1.050)	0.940
Neurological disease	5.855 (3.387–10.122)	< .001
Arterial hypertension	4.236 (2.631–6.821)	< .001
Neoplastic disease	4.575 (2.413–8.672)	< .001
Diabetes	2.950 (1.528–5.696)	0.001
Chronic obstructive pulmonary disease	2.497 (1.291–4.830)	0.007
Moderate-to-severe renal failure	5.835 (2.231–10.088)	0.001
Charlson comorbidity index	1.600 (1.408–1.819)	< .001
Institutionalization	18.851 (4.275–83.135)	< .001

Results are reported as Odds Ratio (OR); 95% Confidence Interval is reported in brackets. P-value: statistical significance

**Fig. 1:**
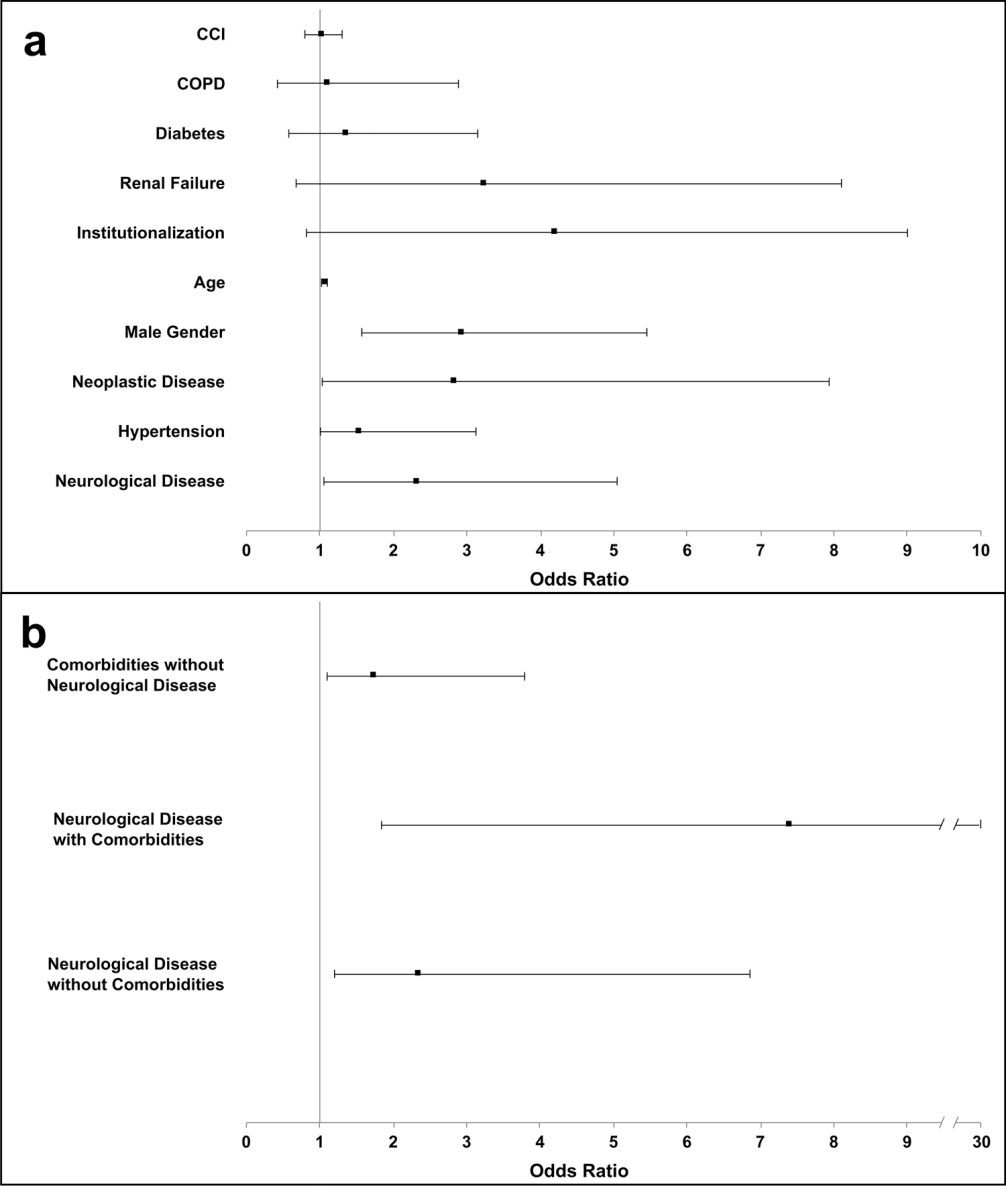
Multivariate analysis of association between patients’ characteristics and severe form of infection. Odds ratios of associations between patients’ characteristics and severe form of infection. In part b, values are adjusted for age and CCI, and patients without any comorbidities were used as reference group. *CCI* Charlson Comorbidity Index, *COPD* chronic obstructive pulmonary disease

On the multivariate binary logistic regression analysis (Fig. 1a), the presence of neurological diseases remained independently associated with severe infection (OR 2.305; 95% CI 1.053–5.046; *p *= 0.012), as well as male gender (*p *= 0.001), older age (*p *= 0.001), neoplastic diseases (*p *= 0.039), and arterial hypertension (*p *= 0.045).

After the ER admittance, patients affected by neurological diseases showed a lower rate of discharge at home (15.6% vs. 42.7%; p < 0.001), a higher rate of non-invasive mechanical respiratory support (15.6% vs. 4.9%; *p *= 0.001) and intensive care indication (14.3% vs. 4.5%; *p *= 0.002). In contrast, they showed a similar rate of hospitalization without the need of mechanical respiratory support (54.5% vs. 47.9%; *p *= 0.307).

After dividing the sample in patients without any comorbidities, patients with neurological disease without other comorbidities, patients with other comorbidities without neurological diseases, and patients with neurological disease and other comorbidities, the latter group showed the strongest association with a severe COVID-19 (OR 7.394; 95% CI 1.840–29.704; *p *= 0.005), compared with patients with neurological (OR 2.332; 95% CI 1.202–6.858; *p *= 0.035) or non-neurological (OR 1.724; 95% CI 1.100–3.790; *p *= 0.041) comorbidity alone (Fig. 1b).

### Comparison of infection severity among different neurological diseases

The association between neurological comorbidity and COVID-19 severity varied among the different neurological diseases (Table 6). Patients affected by cerebrovascular diseases and cognitive impairment showed a higher prevalence of severe infection, a lower rate of discharge at home and a higher rate of non-invasive mechanical ventilation or intensive care indication, significantly different from patients without neurological diseases (p < 0.001). Conversely, patients affected by headache/facial pain, epilepsy, and peripheral neuropathy did not show any significant differences.

**Table 6:** Differences among neurological diseases

	Cerebrovascular (*N *= 30)	Cognitive impairment (*N *= 25)	Headache (N = 11)	Epilepsy (N = 5)	PNP (N= 4)	Patients without neurological disease (N = 267)	*p* value
Severe infection—*N* (%)	20 (66.7%)*	22 (88.0%)*	3 (27.3%)	2 (40.0%)	2 (50.0%)	67 (25.1%)	< .001
Discharge from emergency room—*N* (%)							< .001
Home	4 (13.3%)*	0 (0.0%)*	7 (63.6%)	1 (20.0%)	0 (0.0%)	114 (42.7%)	
Internal Medicine Unit without mechanical respiratory support	16 (53.3%)	15 (60.0%)	3 (27.3%)	3 (60.0%)	3 (75.0%)	128 (47.9%)	
Internal Medicine Unit with non-invasive mechanical respiratory support	3 (10.0%)	7 (28.0%)*	1 (9.1%)	1 (20.0%)	0 (0.0%)	13 (4.9%)	
Intensive Care Unit	7 (23.3%)*	3 (12.0%)	0 (0.0%)	0 (0.0%)	1 (25.0%)	12 (4.5%)	

Results are reported as absolute values (percentage). *p*-value: statistical comparison among groups. *: significant difference vs. Patients without neurological disease (*p* < 0.001). Headache: migraine or chronic tension-type headache or trigeminal neuralgia

Both patients affected by multiple sclerosis and Parkinson disease (not shown in Table 6) suffered from severe COVID-19 that required hospitalization, without the need of mechanical respiratory support.

## Discussion

In this study, we evaluated the prevalence of neurological pre-existing comorbidities in a large cohort of patients admitted to ER and diagnosed with COVID-19, estimating their association with infection severity. Over 20% of patients presented with neurological comorbidities, with cerebrovascular disease and cognitive impairment being the most frequent. Patients with neurological comorbidity showed an OR of 2.3 of suffering from severe COVID-19, even after including age and other clinical and demographic characteristics in the multivariate analysis. This association was stronger when patients suffering from a neurological condition in association with other comorbidities were compared to patients with isolated neurological or non-neurological diseases. Cerebrovascular diseases and cognitive impairment showed higher rate of severe infection and respiratory support indication, and lower rate of discharge at home.

To date, the incidence of new-onset neurological symptoms or syndromes associated with COVID-19 has been reported [3–8], but the description of the relationship between pre-existing neurological comorbidities and infection severity still lacks. A recent review by Herman and colleagues reported a pooled prevalence of neurological comorbidities of 8% among 22 reviewed studies (range 0–40%), without conclusive data on the association with infection severity [12]. A recent study on inpatients from a neurological ward [11], showed worse clinical and functional outcomes, a more frequent use of high-flow oxygenation and antibiotic/antiviral treatments, longer hospitalization, and higher in-hospital mortality rate in patients with neurological diseases and COVID-19, compared to patients without infection; the vast majority of patients with COVID-19 were hospitalized for acute cerebrovascular events. Moreover, a meta-analysis reported that pre-existing cerebrovascular diseases could be an independent risk factor for COVID-19 [15]. In small-sample studies, cerebrovascular diseases have been associated with more frequent ICU admission [16] and with more severe forms of infection [17, 18]. In addition, Du and colleagues reported a 2.4-fold higher mortality risk in patients with cerebrovascular or cardiovascular disease, without specifying the distinction between these two conditions [19].

Our data show that patients with pre-existing cerebrovascular diseases are more frequently hospitalized, needing ICU admission in over 20% of cases, and present with a severe infection in two-thirds of cases. We observed similar findings in cognitively impaired/demented patients. A recent paper reported a prevalence of 6.8% of dementia in a sample of 355 Italian patients who died with COVID-19, without assessing the association with infection severity [20].

In our samples, we observed that patients suffering from neurological diseases were older, more affected by other comorbidities, and more institutionalized. Age is a frequently reported risk factor for severe COVID-19 [16, 19], as well as hypertension [21], diabetes [21, 22], neoplastic diseases [21, 23], COPD [21], and institutionalization [24]. These observations were confirmed in our study. The multivariate analysis showed that even correcting for age, institutionalization, and comorbidities, the presence of neurological diseases seems to be independently associated with a more severe form of infection. The strongest association with severe COVID-19 was observed in patients with both neurological diseases and other comorbidities. This finding is consistent with the results reported by Guan and colleagues, who found hazard ratios for worse infection outcomes as higher as the number of concomitant diseases increases [21]. On the other hand, institutionalization, diabetes, renal failure, and COPD showed a high association with infection severity only in the univariate analysis, but not in the multivariate analysis.

The higher prevalence of severe COVID-19 in patients affected by neurological diseases is probably multifactorial. The intrinsic frailty of chronic and often degenerative conditions, the older age, and the higher comorbidity burden observed in these patients could certainly explain this association [25]. Patients affected by neurological diseases could present a lower ability to compensate for COVID-19, resulting in more severe infection and higher need of ER assistance. Moreover, the potential neurotropism of SARS-CoV-2, with a possible detrimental effect on pre-existing neurological diseases, should also be taken into account [9, 10], as already postulated during the SARS-CoV epidemic in 2003 [26]. While waiting for more accurate pathological evidences, our data underline that patients with neurological diseases, in particular when associated with other comorbidities, represent a population at high risk for severe COVID-19, needing a careful health surveillance.

Our study presents with some limitations. First, the single-center design, which partially restricts the generalizability of our findings. Second, only a minority of patients suffered from neurological diseases without other comorbidities, limiting the conclusions on the association between neurological pathology alone and severe COVID-19. Third, the observations on the prevalence of each neurological disorder, and thus the ability to discriminate disease-specific associations with COVID-19, are limited by two factors: a) the evaluation of patients admitted to ER, which could represent a selection bias towards older people, and b) the lack of adjustments for the relative prevalence of each neurological disease in the general population.

In conclusion, our study reports the prevalence of different neurological diseases in a large cohort of patients with COVID-19, assessing their association with the infection severity. In our sample, patients with pre-existing neurological diseases showed a significantly higher risk for severe infection, in particular when associated with other comorbidities, suggesting that this population deserves a thorough evaluation since the earliest phases of overt or suspected COVID-19. Finally, our findings suggest a particular attention in targeting patients with neurological diseases for proactive viral screening.

## Data Availability

A. Romagnolo had full access to all the data in the study and takes responsibility for the integrity of the data and the accuracy of the data analysis.
